# Comparison of the oxidative potential of primary (POA) and secondary (SOA) organic aerosols derived from α-pinene and gasoline engine exhaust precursors

**DOI:** 10.12688/f1000research.15445.2

**Published:** 2019-03-11

**Authors:** Christopher Lovett, Mohamad Baasiri, Khairallah Atwi, Mohammad H. Sowlat, Farimah Shirmohammadi, Alan L. Shihadeh, Constantinos Sioutas

**Affiliations:** 1 Department of Civil and Environmental Engineering, University of Southern California, Los Angeles, CA, 90089, USA; 2 Department of Mechanical Engineering, American University of Beirut, Riad El Solh, Beirut, 1107 2020, Lebanon

**Keywords:** Particulate Matter, SOA, Biogenic PM, Anthropogenic PM, Photochemical Aging

## Abstract

**Background:** Primary (POA) and secondary (SOA) organic aerosols, deriving from both anthropogenic and biogenic sources, represent a major fraction of ambient particulate matter (PM) and play an important role in the etiology of respiratory and cardiovascular diseases, largely through systemic inflammation and cellular oxidative stress. The relative contributions of these species to the inhalation burden, however, are rather poorly characterized. In this study, we measured the *in vitro* oxidative stress response of alveolar macrophages exposed to primary and secondary PM derived from both anthropogenic and biogenic sources.

**Methods:** POA and SOA were generated within an oxidation flow reactor (OFR) fed by pure, aerosolized α-pinene or gasoline engine exhaust, as representative emissions of biogenic and anthropogenic sources, respectively. The OFR utilized an ultraviolet (UV) lamp to achieve an equivalent atmospheric aging process of several days.

**Results:** Anthropogenic SOA produced the greatest oxidative response (1900 ± 255 µg-Zymosan/mg-PM), followed by biogenic (α-pinene) SOA (1321 ± 542 µg-Zymosan/mg-PM), while anthropogenic POA produced the smallest response (51.4 ± 64.3 µg-Zymosan/mg-PM).

**Conclusions:** These findings emphasize the importance of monitoring and controlling anthropogenic emissions in the urban atmosphere, while also taking into consideration spatial and seasonal differences in SOA composition. Local concentrations of biogenic and anthropogenic species contributing to the oxidative potential of ambient PM may vary widely, depending on the given region and time of year, due to factors such as surrounding vegetation, proximity to urban areas, and hours of daylight.

## Introduction

A large fraction of ambient particulate matter (PM) in the urban atmosphere consists of a mixture of primary organic aerosols (POA), derived from anthropogenic and biogenic PM sources, as well as secondary organic aerosols (SOA) produced during the photo-oxidation of both types of POA ( Baltensperger *et al.*, 2005; Després *et al.*, 2012). Urban PM can consist of up to 90% SOA, the majority originating from primary biogenic aerosols, including the monoterpene α-pinene, one of the largest components of primary biogenic PM worldwide ( Hallquist *et al.*, 2009; Seinfeld & Pankow, 2003).

Several human health problems linked to ambient PM, including asthma, cardiovascular disease, and heart failure ( Delfino *et al.*, 2005; Dominici *et al.*, 2006; Kim *et al.*, 2013; Shah *et al.*, 2013), are mediated largely by the cellular inflammatory response, including reactive oxygen species (ROS) formation ( Li *et al.*, 2003; Ray *et al.*, 2012). Research investigating PM health effects has mostly focused on primary emissions, while studies of secondary PM effects are not as common. Some studies, however, report that both anthropogenic ( Decesari *et al.*, 2017; Saffari *et al.*, 2015; Verma *et al.*, 2014; Verma *et al.*, 2015a; Verma *et al.*, 2015b) and biogenic ( Baltensperger *et al.*, 2008; Gaschen *et al.*, 2010; Rohr, 2013) SOA elicit greater adverse health effects than POA precursors.

In this study, we investigate the effects of photochemical oxidation on the oxidative potential of biogenic and anthropogenic PM. Samples of each PM type were collected before and after photochemical aging within a laboratory reaction chamber equipped with an ultraviolet lamp. The *in vitro* alveolar macrophage (AM) assay was used to quantify PM oxidative potential ( Landreman *et al.*, 2008; Li *et al.*, 2008; Shafer *et al.*, 2010).

## Methods

### Sampling methods

Photochemical oxidation of primary emissions occurred within a 64-liter stainless steel oxidation flow reactor (OFR) equipped with a single UV lamp (BHK Analamp, Model No. 82-9304-03) emitting radiation at 185 and 254 nm. Upstream of the PM sources, inlet air first passed through an activated carbon denuder and high-efficiency particulate air (HEPA) filter to remove all particles. Within the OFR, a warm, humid environment (22°C/60% RH) was maintained, allowing H _2_O to act as a source of hydroxyl radicals in the UV-catalyzed oxidation reactions, which resulted in SOA formation. While the production of superoxide radicals is possible, the major products of H _2_O oxidation generated in this type of OFR are hydroxyl radicals, especially at less than 80% relative humidity ( Seinfeld & Pandis, 2016; Kamens *et al.,* 2011; Jang *et al.,* 2002).

**Figure 1.**
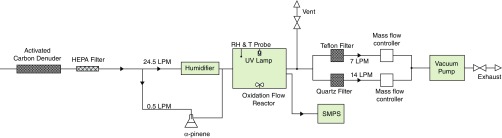
Biogenic (α-pinene) particulate matter (PM) sampling setup.

The biogenic sampling setup is depicted in Figure 1. Particle-free inlet air was introduced at a flow rate of 25 lpm. 0.5 lpm of this incoming air stream was diverted into a 250 ml Büchner flask containing a 15-ml glass vial of pure, reagent grade α-pinene. Three small holes in the vial cap allowed for diffusion of α-pinene vapors into the flask. The remaining 24.5 lpm flow of particle-free air proceeded through a humidifier (heated flask containing distilled water) and into the reactor, where it mixed with the α-pinene vapors, resulting in a dilution ratio of 50:1.

**Figure 2.**
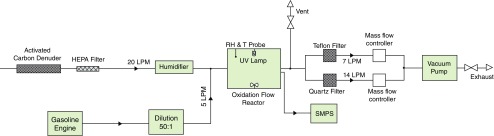
Anthropogenic (gasoline engine exhaust) particulate matter (PM) sampling setup.

The anthropogenic sampling setup is depicted in Figure 2. Exhaust from a four-stroke single cylinder gasoline generator (Honda SHX1000, 49cc displacement, 8.0:1 compression ratio, operating at 3000 RPM) was drawn through a rotating disk dilutor (RDD; Testo Engineering, MD19-3E) operating at a dilution ratio of 50:1. 5 lpm of the diluted engine exhaust was diverted into the reaction chamber, where it mixed with 20 lpm of humidified, particle-fee air, resulting in a total dilution ratio of 250:1.

Particles were collected downstream of the reaction chamber on Teflon and quartz filters. In the POA condition, PM was collected as the α-pinene or engine emissions passed through a dark OFR. In the SOA condition, the aerosol stream was sampled while the UV lamp inside the reactor was on. During this condition, the sample stream was diverted for the first 90 minutes to ensure that the reactor was operating under steady-state conditions while samples were collected.

### Filter conditioning

Prior to sampling, quartz filters were baked in a furnace oven at 500°C for 5 hours. Teflon filters were conditioned for 24 hours in a controlled environment (23°C and 46% relative humidity) before weighing. Teflon filters were weighed before and after sampling to determine the mass collected with an MT5 Microbalance (Mettler-Toledo Inc., Columbus, OH, USA). Mass collected on quartz filters was calculated based on the aerosol concentration (from Teflon filters) and sampling flow rate. After sampling, all filters were placed in petri dishes lined with baked aluminum foil, sealed with Teflon tape, and stored in a refrigerated environment until analysis.

### Laboratory analyses

Quartz filters were analyzed for elemental carbon (EC) and organic carbon (OC) content by the National Institute for Occupational Safety and Health (NIOSH) Thermal Optical Transmission (TOT) Method 5040, using a flame ionization detector (FID) to quantify evolved carbon as CH _4_ ( Birch & Cary, 1996; Peterson & Richards, 2002).

Each Teflon filter (PM sample or blank) was divided into two sections, one for the macrophage assay and one for chemical analysis. The filter halves used for the cell assay underwent sonication in 900 μL of purified water for approximately 16 hours at room temperature to extract the water-soluble components. These filters were then removed from the aqueous extracts, and 10x concentrated salts-glucose medium (SGM) was added to create buffered PM extract solutions for use in the PM treatments.

The alveolar macrophage (AM) *in vitro* assay was used to determine the oxidative potential of the Teflon filter PM samples. Alveolar macrophages obtained from the American Type Culture Collection (cell line NR8383, RRID: CVCL_4396) were maintained in Ham’s F12 medium (#11765-047, ThermoFisher, Waltham, MA, USA) supplemented with 2mM L-glutamine (GlutaMAX; #31765-035, ThermoFisher, Waltham, MA, USA), 1.176 g/L sodium bicarbonate, and 15% heat inactivated fetal bovine serum (FBS; #45000-734, VWR, Radnor, PA, USA). Cells were cultured in flasks and kept in an incubator at 37°C/5% CO _2_. Non-adherent cells were transferred to new flasks weekly. A floating cell concentration of approximately 4 × 10 ^5^ cells/mL media was maintained.

In preparation for PM exposures, the non-adherent fraction of macrophage cells was harvested from flasks and concentrated by centrifugation (750 RPM) for 5 minutes. The culture medium was removed, and the cell suspension was diluted to a concentration of 1,000 cells/μL. 100 μL aliquots of these suspended cells were then pipetted into each well of 96-well plates (100,000 cells/well) and incubated at 37°C/5% CO _2_ for 2 hours. Following the 2-hour incubation period, nearly all macrophages were adhered to each well bottom. During PM treatments, supernatant media was aspirated and replaced with 100 μL of sample extract or blank control solution in each well.

The macrophage cells were exposed to each type of PM sample for 2.5 hours, using 2,7-dichlorodihydrofluorescein diacetate (DCFH-DA) as a fluorescent probe to quantify the cellular formation of oxidative species. The non-fluorescent DCFH-DA acts by entering the cell, where it is de-acetylated by cellular enzymes to yield 2,7-dichlorodihydrofluorescein (DCFH), also non-fluorescent. DCFH is then oxidized by reactive species, generated during the cellular oxidative stress response to PM exposure, to form the highly fluorescent and detectable 2,7-dichlorofluorescein (DCF), which was quantified spectrophotometrically with a CytoFlour II automated fluorescence plate reader (PerSeptive Biosystems, Framingham, MA, USA) Each treatment or control was run in triplicate (3 wells each). Additionally, several untreated and method blank controls were included on each 96-well plate. Zymosan was included as a positive control at a concentration of 0.125 mg-zymosan/mL in a buffered SGM solution. Results are reported as the increase in fluorescence due to sample treatments relative to the fluorescence observed in the untreated control condition ( Landreman *et al.*, 2008; Shafer *et al.*, 2010).

## Results

**Figure 3.**
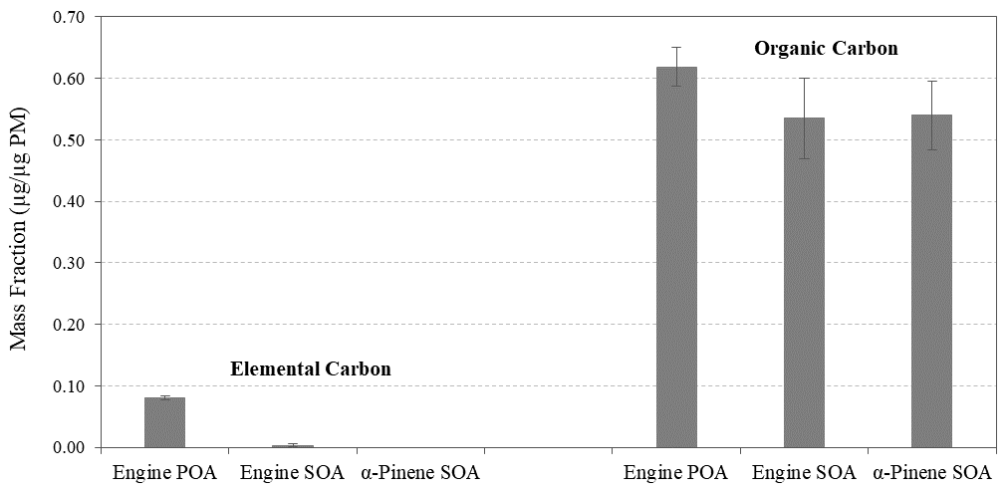
Mass fractions of elemental carbon (EC) and organic carbon (OC) in engine primary organic aerosol (POA) and engine & α-pinene secondary organic aerosols (SOA). Error bars represent laboratory uncertainty values based on contributions of analytical error (standard deviation) and blank subtraction (standard deviation of at least three method blanks).

**Figure 4.**
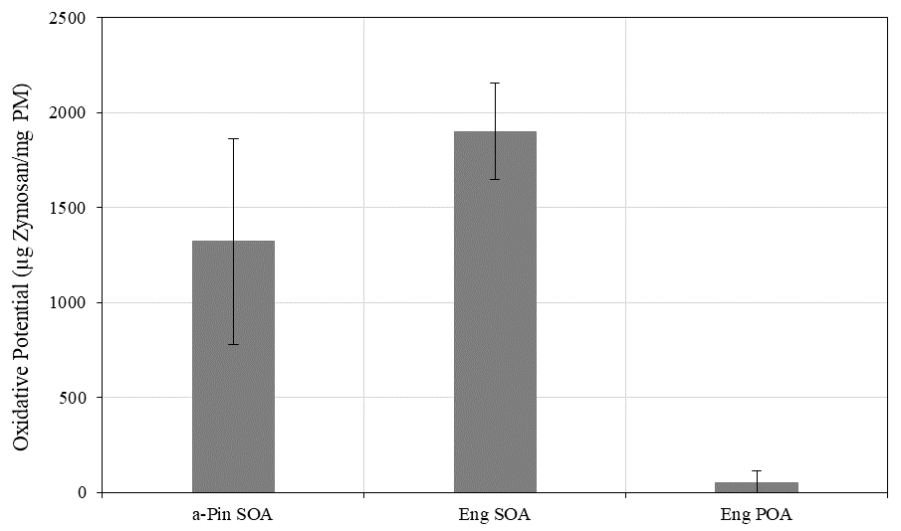
Mass-fraction based oxidative potential: Engine primary organic aerosol (POA) and engine & α-pinene secondary organic aerosols (SOA). Error bars represent laboratory uncertainty values based on contributions of analytical error (standard deviation) and blank subtraction (standard deviation of at least three method blanks).

EC/OC results are presented in Figure 3. EC was most abundant in engine POA (0.081 μg-EC/μg-PM), with no significant amount present in either engine or α-pinene SOA. Mass fractions of OC were higher than EC in all conditions (engine POA: 0.62 μg-OC/μg-PM, engine SOA: 0.54 μg-OC/μg-PM, α-pinene SOA: 0.54 μg-OC/μg-PM). Figure 4 presents oxidative potential results on a mass-fraction basis, standardized to Zymosan units (μg-Zymosan units/mg-PM). Mass fraction results reveal how the intrinsic PM toxicity as indexed by oxidative potential changes over time due to photochemical aging. The measured oxidative potential for engine POA was 51.4 (± 64.3) µg-Zymosan/mg-PM, and for engine SOA it was 1900 (± 255) µg-Zymosan/mg-PM, while for α-pinene SOA, the result was 1321 (± 542) µg-Zymosan/mg-PM (pure α-pinene was not assayed).

Figure 3 EC-OC Raw Data Click here for additional data file. Copyright: © 2019 Lovett C et al. 2019 Data associated with the article are available under the terms of the Creative Commons Zero "No rights reserved" data waiver (CC0 1.0 Public domain dedication).

Figure 4 ROS Raw Data Click here for additional data file. Copyright: © 2019 Lovett C et al. 2019 Data associated with the article are available under the terms of the Creative Commons Zero "No rights reserved" data waiver (CC0 1.0 Public domain dedication).

## Summary and conclusions

The findings of the current reaction chamber study indicate that both anthropogenic and biogenic SOA induce greater cellular oxidative stress than primary engine exhaust. This effect was found to be largest in response to engine exhaust SOA, thus implicating anthropogenic PM as the major contributor to adverse human health effects in urban environments, though the contribution of biogenic SOA can be quite significant in some geographical areas. Atmospheric aging of PM increases its intrinsic oxidative potential many fold, and thus photochemistry in a region that experiences abundant sunshine, long days, and/or stagnation of circulating air due to an inversion layer or some other reason, may increase the toxicity of PM over time.

While the macrophage assay is generally more sensitive to water-soluble components of PM, as compared to non-polar PM species, the large cellular oxidative stress response to SOA, especially anthropogenic SOA, cannot be attributed entirely to hydrophilicity. Biogenic organic compounds are generally more hydrophilic than organic products of anthropogenic processes such as combustion, yet greater ROS activity in response to anthropogenic PM was observed. The alveolar macrophage assay is considered an excellent model of inhalation toxicity, and increased ROS formation reliably indexes greater toxicity, whether ultimately due to increased bioavailability of harmful molecules or simply greater quantities of harmful PM species ( Landreman *et al.*, 2008). Thus, the results of this study clearly indicate that oxidized PM species, whether of biogenic or anthropogenic origin, are more toxic that primary PM, regardless of water-solubility, with the greatest toxicity resulting from anthropogenic SOA exposures.

## Data availability

The data referenced by this article are under copyright with the following copyright statement: Copyright: © 2019 Lovett C et al.

Data associated with the article are available under the terms of the Creative Commons Zero "No rights reserved" data waiver (CC0 1.0 Public domain dedication). 

The following raw data sets are provided as comma separated values (.csv) files:

Dataset 1: Figure 3 EC-OC Raw Data 10.5256/f1000research.15445.d209280 ( Lovett *et al.*, 2018a)

Dataset 2: Figure 4 ROS Raw Data 10.5256/f1000research.15445.d209281 ( Lovett *et al.*, 2018b)
