# Supplementary material for: Comparison of the oxidative potential of primary (POA) and secondary (SOA) organic aerosols derived from α-pinene and gasoline engine exhaust precursors
Source: F1000Res. 2019 Mar 11;7:1031. Originally published 2018 Jul 9. [Version 2] doi: 10.12688/f1000research.15445.2 (PMC6392154; doi:10.12688/f1000research.15445.2)
Supplement: Peer review history

# Peer review: Reviewer response for version 1

**DOI:** 10.5256/f1000research.16832.r43635

**Published:** 2019-02-25

**Authors:** Zhi Ning

**Affiliations:** 1 Division of Environment and Sustainability, Hong Kong University of Science & Technology, Hong Kong, China

**General Comments:**

 The authors have investigated the oxidative potential of POA and SOA from two different sources namely alpha-pinene and gasoline engine exhaust. The experimental setup included an UV chamber (oxidation flow reactor), to mimic the sun light’s UV rays, to compare primary and secondary organic aerosol-induced radical generation under light and in dark. The comparison could contribute great value to the manuscript if additional parameters as listed below are included in it:

1. Page 3: Right column: Line 4: The authors can address why they have selected only Hydroxyl radicals in the investigations. In some experiments, where UV rays are used to excite the organic aerosols, the elicitation of superoxide radicals is also possible.
2. Page 3: Right column: Lines 28-30: The statement “aerosol stream was sampled while a UV lamp was on, following a 90-minute reaction period.” is not clear. Does that mean the whole sample streaming is done for a continuous 90 minutes? Was it the same for the aerosol sampling done in dark OFR?
3. Page 3: Right column: Line 33: The information of the control sample needs to be included here.
4. Page 4: Left column: Line 23: The analysis part has some information missing such as incubation time for cell growth, and are the same generation (life cycle) used for analysis?
5. Page 4: Left column: Line 25: The cell exposure study has some basic information missing - PM dose, route of exposure (directly on filters or on PM extracts), number of times analysed (duplicate or triplicate). Please include for clarity.
6. Page 5: Figure 4: The biogenic organic compounds (for example: the alpha-pinene) are believed to be more hydrophilic compared to engine exhaust organics. Please include a discussion if the water solubility of samples is also driving the difference in oxidative stress.

I have read this submission. I believe that I have an appropriate level of expertise to confirm that it is of an acceptable scientific standard.

# Response

**Published:** 2019-03-05

**Authors:** Christopher Lovett

**Affiliations:** University of Southern California, USA

Authors’ responses to specific comments of Dr. Zhi Ning:

Comment 1: *Page 3: Right column: Line 4: The authors can address why they have selected only Hydroxyl radicals in the investigations. In some experiments, where UV rays are used to excite the organic aerosols, the elicitation of superoxide radicals is also possible.*

**Text Added:**

“While the production of superoxide radicals is possible, the major products of H _2_O oxidation generated in this type of OFR are hydroxyl radicals, especially at less than 80% relative humidity (Seinfeld & Pandis, 2016; Kamens et al., 2011; Jang et al., 2002).”

Comment 2: *Page 3: Right column: Lines 28-30: The statement “aerosol stream was sampled while a UV lamp was on, following a 90-minute reaction period.” is not clear. Does that mean the whole sample streaming is done for a continuous 90 minutes? Was it the same for the aerosol sampling done in dark OFR?*

**Text Revision:**

“In the SOA condition, the aerosol stream was sampled while the UV lamp inside the reactor was on. During this condition, the sample stream was diverted for the first 90 minutes to ensure that the reactor was operating under steady-state conditions while samples were collected.” 

Comment 3: *Page 3: Right column: Line 33: The information of the control sample needs to be included here.*

**Authors’ Response:**

The only control samples were the filter blanks submitted to the laboratory for chemical and toxicological analyses as discussed in Section 2.3. Discussion of the control condition utilized in the macrophage assay (untreated cells) is included in the response to Comment 5. 

Comment 4: *Page 4: Left column: Line 23: The analysis part has some information missing such as incubation time for cell growth, and are the same generation (life cycle) used for analysis?*

**Text Added:**

“In preparation for PM exposures, the non-adherent fraction of macrophage cells was harvested from flasks and concentrated by centrifugation (750 RPM) for 5 minutes. The culture medium was removed, and the cell suspension was diluted to a concentration of 1,000 cells/μL. 100 μL aliquots of these suspended cells were then pipetted into each well of 96-well plates (100,000 cells/well) and incubated at 37°C/5% CO _2_ for 2 hours. Following the 2-hour incubation period, nearly all macrophages were adhered to each well bottom. During PM treatments, supernatant media was aspirated and replaced with 100 μL of sample extract or blank control solution in each well.” 

Comment 5: *Page 4: Left column: Line 25: The cell exposure study has some basic information missing - PM dose, route of exposure (directly on filters or on PM extracts), number of times analysed (duplicate or triplicate). Please include for clarity.*

**Text Added:**

“Each Teflon filter (PM sample or blank) was divided into two sections, one for the macrophage assay and one for chemical analysis. The filter halves used for the cell assay underwent sonication in 900 μL of purified water for approximately 16 hours at room temperature to extract the water-soluble components. These filters were then removed from the aqueous extracts, and 10x concentrated salts-glucose medium (SGM) was added to create buffered PM extract solutions for use in the PM treatments.”“Each treatment or control was run in triplicate (3 wells each). Additionally, several untreated and method blank controls were included on each 96-well plate. Zymosan was included as a positive control at a concentration of 0.125 mg-zymosan/mL in a buffered SGM solution. Results are reported as the increase in fluorescence due to sample treatments relative to the fluorescence observed in the untreated control condition.”

Comment 6: *Page 5: Figure 4: The biogenic organic compounds (for example: the alpha-pinene) are believed to be more hydrophilic compared to engine exhaust organics. Please include a discussion if the water solubility of samples is also driving the difference in oxidative stress.*

**Text Added (to Summary and Conclusions):**

“While the macrophage assay is generally more sensitive to water-soluble components of PM, as compared to non-polar PM species, the large cellular oxidative stress response to SOA, especially anthropogenic SOA, cannot be attributed entirely to hydrophilicity. Biogenic organic compounds are generally more hydrophilic than organic products of anthropogenic processes such as combustion, yet greater ROS activity in response to anthropogenic PM was observed. The alveolar macrophage assay is considered an excellent model of inhalation toxicity, and increased ROS formation reliably indexes greater toxicity, whether ultimately due to increased bioavailability of harmful molecules or simply greater quantities of harmful PM species (Landreman et al., 2008). Thus, the results of this study clearly indicate that oxidized PM species, whether of biogenic or anthropogenic origin, are more toxic that primary PM, regardless of water-solubility, with the greatest toxicity resulting from anthropogenic SOA exposures.”

# Peer review: Reviewer response for version 1

**DOI:** 10.5256/f1000research.16832.r39486

**Published:** 2018-11-06

**Authors:** Barend L. van Drooge

**Affiliations:** 1 Institute for Environmental Assessment and Water Research (IDÆA-CSIC), Barcelona, Spain

The work of Lovett *et al.* presents interesting data on the possible inflammatory effects of SOA from traffic as well as biogenic (pinene) origin. The method setup is well designed, although the variables, such as conditions of relative humidity and temperature, could have been studied in different values. On the other hand, the results show that the biogenic SOA have similar high inflammatory effect in the test compared to traffic SOA, which is an important fact, and indicates that effects have been observed in real-world data. However, the study could have been more complete and higher quality if the researchers would have made an effort to detect and quantify the molecular chemical compounds that are present in the SOA fractions. 

 The work is suitable for indexing.

I have read this submission. I believe that I have an appropriate level of expertise to confirm that it is of an acceptable scientific standard.
